# Modelling midline shift and ventricle collapse in cerebral oedema following acute ischaemic stroke

**DOI:** 10.1371/journal.pcbi.1012145

**Published:** 2024-05-28

**Authors:** Xi Chen, Tamás I. Józsa, Danilo Cardim, Chiara Robba, Marek Czosnyka, Stephen J. Payne

**Affiliations:** 1 Institute of Biomedical Engineering, Department of Engineering Science, University of Oxford, Oxford, United Kingdom; 2 School of Aerospace, Transport and Manufacturing Cranfield University, Cranfield, United Kingdom; 3 Department of Neurology, University of Texas Southwestern Medical Centre, Dallas, Texas, United States of America; 4 Institute for Exercise and Environmental Medicine, Texas Health Presbyterian Hospital, Dallas, Texas, United States of America; 5 Department of Anesthesia and Intensive Care, IRCCS Ospedale Policlinico San Martino, Genova, Italy; 6 Division of Neurosurgery, Department of Clinical Neurosciences, University of Cambridge, Cambridge, United Kingdom; 7 Institute of Electronic Systems, Warsaw University of Technology, Warsaw, Poland; 8 Institute of Applied Mechanics, National Taiwan University, Taiwan; Stanford University, UNITED STATES

## Abstract

In ischaemic stroke, a large reduction in blood supply can lead to the breakdown of the blood-brain barrier and to cerebral oedema after reperfusion therapy. The resulting fluid accumulation in the brain may contribute to a significant rise in intracranial pressure (ICP) and tissue deformation. Changes in the level of ICP are essential for clinical decision-making and therapeutic strategies. However, the measurement of ICP is constrained by clinical techniques and obtaining the exact values of the ICP has proven challenging. In this study, we propose the first computational model for the simulation of cerebral oedema following acute ischaemic stroke for the investigation of ICP and midline shift (MLS) relationship. The model consists of three components for the simulation of healthy blood flow, occluded blood flow and oedema, respectively. The healthy and occluded blood flow components are utilized to obtain oedema core geometry and then imported into the oedema model for the simulation of oedema growth. The simulation results of the model are compared with clinical data from 97 traumatic brain injury patients for the validation of major model parameters. Midline shift has been widely used for the diagnosis, clinical decision-making, and prognosis of oedema patients. Therefore, we focus on quantifying the relationship between ICP and midline shift (MLS) and identify the factors that can affect the ICP-MLS relationship. Three major factors are investigated, including the brain geometry, blood-brain barrier damage severity and the types of oedema (including rare types of oedema). Meanwhile, the two major types (stress and tension/compression) of mechanical brain damage are also presented and the differences in the stress, tension, and compression between the intraparenchymal and periventricular regions are discussed. This work helps to predict ICP precisely and therefore provides improved clinical guidance for the treatment of brain oedema.

## Author summary

Midline shift has been widely employed in clinical settings to estimate the severity of post-stroke brain oedema. However, the midline shift is affected by many factors and its relationship with intracranial pressure has been proven to vary among patients. In this paper, we utilise a computational tool to investigate the midline shift under large brain deformation from a mechanical perspective. The Augmented Lagrange Method is for the first time employed to solve the large deformation of oedema brains with midline shifts over 5 mm. In the model validation, a reasonable agreement between clinical data and simulation results are obtained. This paper models three different factors that can affect midline shift during oedema development, including brain geometry, blood-brain barrier damage severity and type of oedema. Meanwhile, we discuss the scenarios that can lead to unexpectedly small midline shifts and present quantitative results for a better understanding of brain mechanics during oedema.

## 1. Introduction

**Fig 1:**
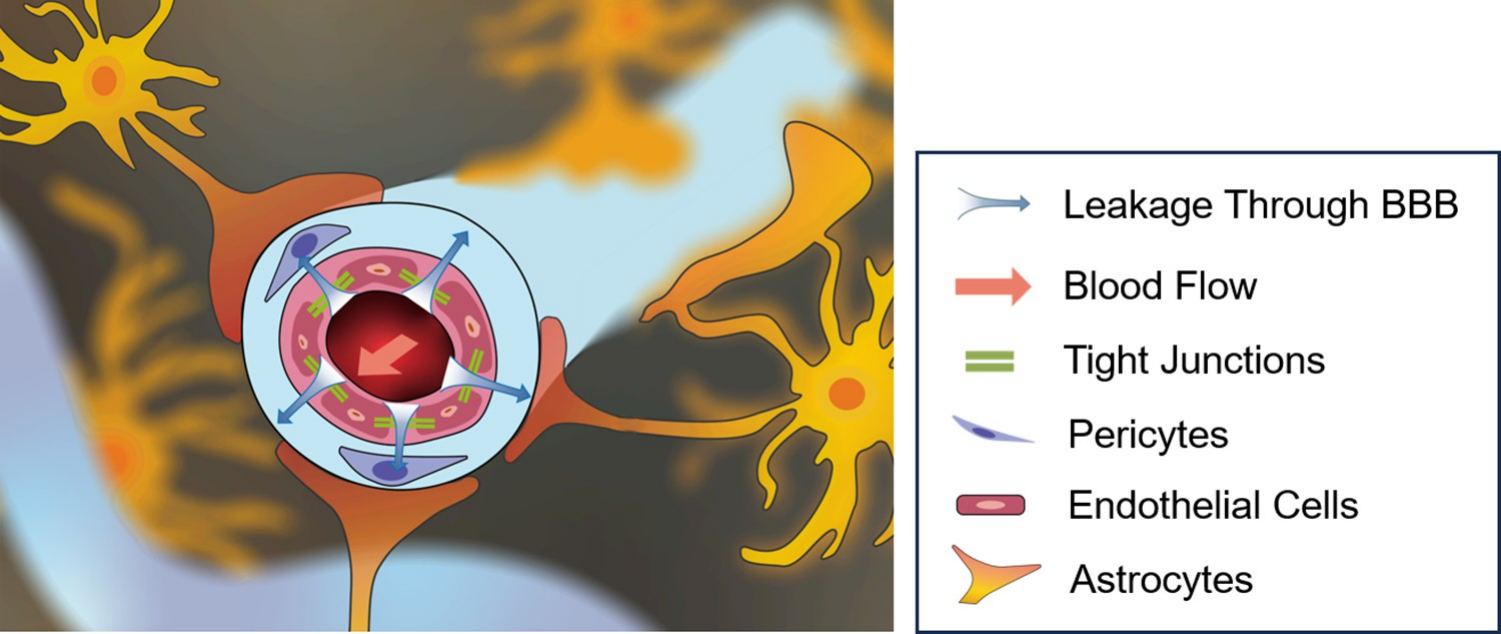
Illustration of blood leaking from a capillary vessel segment. Oedema formation is associated with blood leaving the lumen through the damaged blood brain barrier, filling the interstitial space, and thus causing tissue swelling.

The human brain requires continuous and adequate blood flow to function properly. Following an ischaemic stroke, blood vessel occlusion can reduce cerebral blood flow (CBF), leading to the breakdown of the blood-brain barrier (BBB) and increased BBB permeability [1,2]. Reperfusion therapy restores CBF, but it can also cause excessive fluid leakage, leading to brain oedema and increased ICP (Fig 1) [3]. The fluid accumulation and increased ICP in the brain lead to swelling of the brain tissue. As the oedema core swells and compresses the surrounding tissue, stress and stretch in this tissue rise and result in tissue damage [4]. This can cause disruption in the functioning of different parts of the brain and lead to permanent brain damage or even death.

An elevated ICP can cause symptoms such as headache, nausea, vomiting, and loss of consciousness and can be fatal when over 22 mmHg [5]. Therefore, clinical decision-making and treatment strategies heavily depend on accurate knowledge of ICP levels and generally aim to restore the ICP level to a normal range of 5–15 mmHg [5]. The ICP can be measured invasively in intraventricular, intraparenchymal, subdural and epidural spaces [6]. The gold standard of ICP measurement is through an intraventricular pressure probe [7], but such an invasive technique involves higher risks of brain damage and post-surgery infection [8]. Therefore, multiple non-invasive ICP measurement techniques have been developed to attempt to overcome the limitations of invasive techniques [6,9,10,11]. Many studies have attempted to find a correlation between increased ICP and radiologic findings on CT/MRI images, including the appearance of the cisterns, ventricular size, degree of contusion, the magnitude of MLS, and the ventricular index [12]. However, these criteria are affected by many factors and thus are not able to predict ICP precisely. [11,13].

In this study, we focus on the MLS of the oedematous brain, which is defined as the shift of the brain past its centre line. It has been widely used to estimate ICP and the severity of brain damage since MLS can be observed directly on brain imaging. Meanwhile, MLS is also an important diagnostic [14,15] and prognostic [16] indicator in various brain diseases because it provides important information about the location and severity of brain injury [17,18,19]. Usually, a midline shift of less than 5 mm may be considered mild and may not require immediate intervention, while a midline shift of more than 5 mm may be considered moderate to severe and may require urgent surgical intervention to relieve the pressure [20]. Although previous studies have shown a linear relationship between ICP and MLS, this relationship still lacks statistical significance [13].

Over the past few decades, computational models have been widely applied to study brain oedema caused by various diseases [21–29] and only a few studies have focused on the simulation of post-stroke oedema [24,30]. Most of the previous studies focused on the solid mechanics of the brain tissue and are therefore not able to represent the variation of ICP across the computation domain and reflect the relationship between ICP and MLS [30–32]. In addition, previous studies have only investigated small deformation due to the difficulties in simulating large deformations and ventricle collapse and are thus not able to simulate MLS of over 5 mm [30].

To provide more insights into the ICP-MLS relationship, we propose a new computational model to study the relationship between ICP and MLS and explore the factors that could cause deviations from a linear relation. The model has been utilised for the simulation of brain osmotherapy and haemorrhagic transformation after stroke and validated with clinical data [33,34]. The present study sets out to extend computational brain models developed in the In Silico Clinical Trials for the Treatment of Acute Ischaemic Stroke (INSIST) project [28,35–37] by incorporating a contact mechanics solver to simulate oedema with large deformations and ventricle collapse and then to investigate factors such as patient brain geometry, the severity of BBB damage and types of oedemas that can affect the ICP-MLS relation. Once a reliable model is established, it will help to sharpen the focus of resource-intensive clinical studies, and thus lower the associated (often very high) costs. Inspired by recent studies [24,26,29] on biofluid transport in the brain, a multi-compartment porous Finite Element model will be utilised to investigate the ICP-MLS relation.

In this study, we thus used poroelastic theory to model brain oedema and ventricle collapse and to investigate the relation between ICP and MLS. The computational model incorporates a contact mechanics solver for the first time to investigate severe brain oedema with large deformations and ventricle collapse. The study consists of the following parts: (1) the governing equations will be presented and the simulated arterial blood pressure (ABP), ICP and MLS will be compared with clinical data to validate the model; (2) the simulation results will be presented to show the distribution of ICP, displacement, stress and stretch in the oedematous brain; (3) oedema in patient-specific brain geometries, oedema with various severity of BBB damage and different types of oedema will be investigated to show their effects on the ICP-MLS relationship; (4) the ICP-MLS relations of different cases will be compared and the important factors will be highlighted to improve the clinical estimation of ICP; (5) stress and tension/compression that can lead to brain tissue damage will be discussed.

## 2. Results

**Fig 2:**
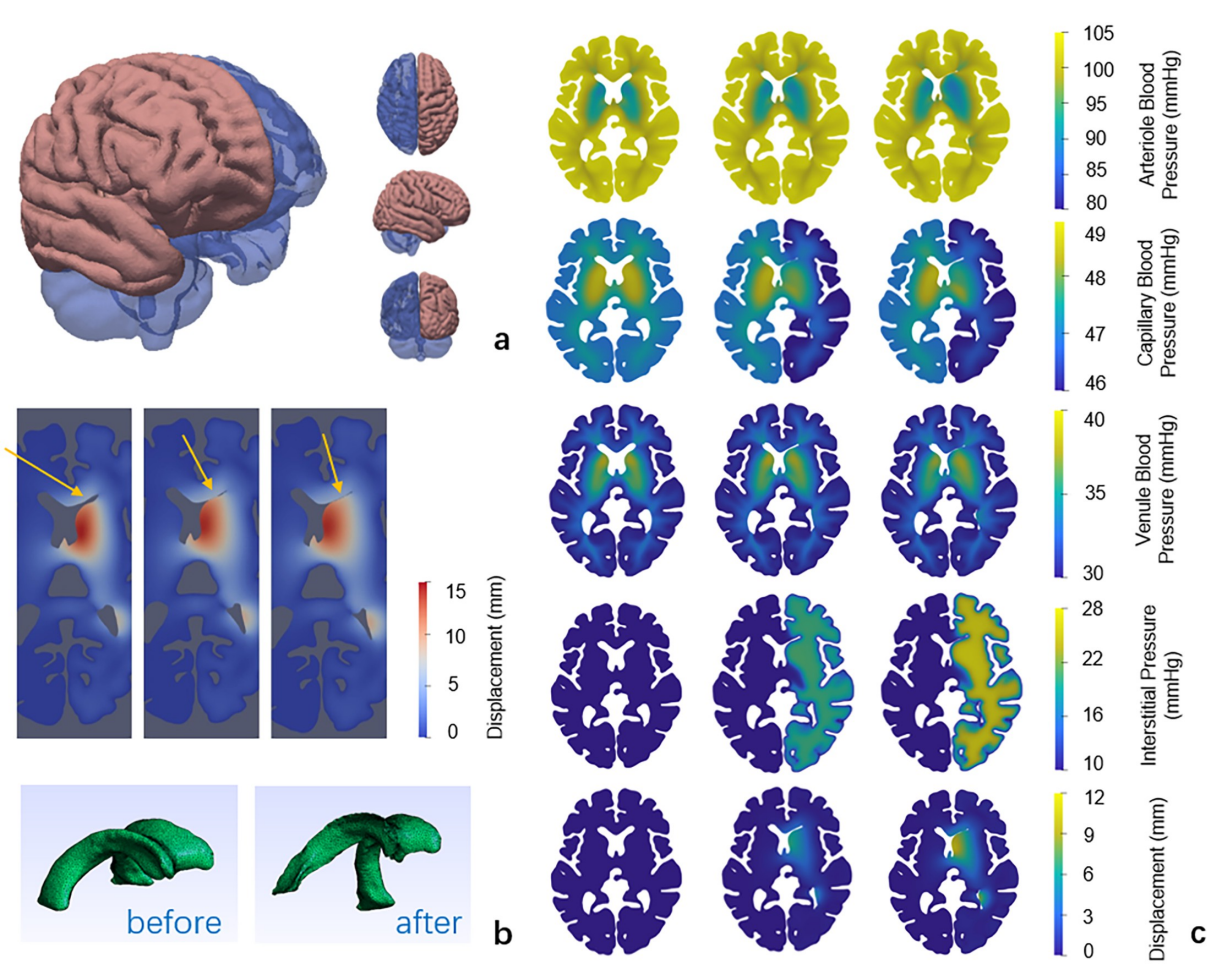
Simulation results of the hemispheric oedema in the population-averaged human brain when the ABP is 105 mmHg. Here, the simulation results for 105 mmHg ABP are chosen to show the MLS and contact of ventricle surface more clearly. (a) 3D hemispheric oedema geometry (pink) and whole brain geometry (blue) (b) Partial contact surfaces (marked by yellow arrows) and ventricle collapse before and after deformation. (c) Slices of blood pressures, ICP and displacement at 0, 500 and 1000 seconds (from left to right column).

Here we show the simulation results of the population-averaged brain [29,38] with hemispheric stroke. The oedema volume is simulated in the perfusion model and shown in Fig 2A. As the brain swells during the development of oedema, the cerebrospinal fluid (CSF) drains, and the ventricle surface contacts (Fig 2B). The temporal variation of blood pressures, ICP and displacement can be observed. As the ICP rises, the brain swells and the capillary blood pressure drops on the swelling side. Meanwhile, there is only an insignificant change in the blood pressures in the arteriole and venule compartments (Fig 2C).

### 2.1 Model validation

**Fig 3:**
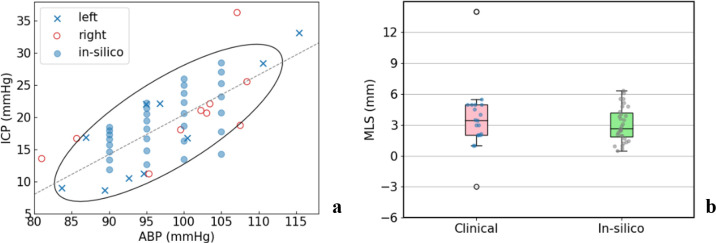
Clinical data and validation. (a) The ICP and ABP relationship with 75% confidence interval ellipse fit and linear fit (dotted line). Clinical data for lesions on the left and right hemisphere are shown and simulations are run within the range. (b) Clinical and in-silico MLS comparison, with the boxplots showing 5, 25, 50, 75, 95 percentiles.

The simulation results are first compared with the clinical ICP, ABP and MLS data of oedema patients. In Fig 3, the clinical data of ICP and ABP in lesions in the left hemisphere and right hemisphere are shown separately, with the in-silico data fitting the ellipse. In the clinical data, a linear relationship between ICP and ABP shows that the ICP rise is primarily driven by blood pressure and the data points can be fitted using a 75% confidence interval ellipse and linear fit (as shown in Fig 3). Similarly, the governing equations in the model also indicate that the ICP rise is linearly driven by the ABP, as shown in the oedema model section. By varying the ABP values, ICP also varies during brain oedema and the ICP values can be recorded and compared with the clinical data to fit the ellipse. In clinical settings, patients can be registered at different times after stroke onset and therefore ICP can be measured at different stages in oedema development. Here, we use different ICP values from various time steps (total time of 1000s and each time step 125s) under a certain ABP value to fit the ellipse. A good fit of the ABP and ICP data indicates a proper choice of the *L*_*p*_ to *K*_*w*_ ratio in the computational model and thus validates the flow simulation. In the model, ABP corresponds to the boundary blood pressure on the pial surface in the arteriole compartment and ICP corresponds to the maximum interstitial pressure across the computation domain. MLS is measured as the maximum displacement on the centreline of the brain model. As for different ABP values *ABP*_*i*_, the boundary conditions for the arteriole blood at the cortical surface are varied and the venule pressure is set to rise as the arteriole pressure rises, with a fixed pressure difference of 75 mmHg.

Meanwhile, we can also obtain the MLS values in the simulations for the validation of the solid compartment in our model. In our model, the relationship between ICP and MLS is determined by the shear modulus and Poisson ratio of the solid phase. Therefore, a comparison demonstrates the accuracy of mechanical property parameters chosen in our model. In Fig 3B, the MLS values of the data points in Fig 3A are plotted against the boxplot of MLS values measured on CT imaging, and the in-silico and clinical data are found to match reasonably well.

### 2.2 Intracranial pressure, midline shift, and stress

**Fig 4:**
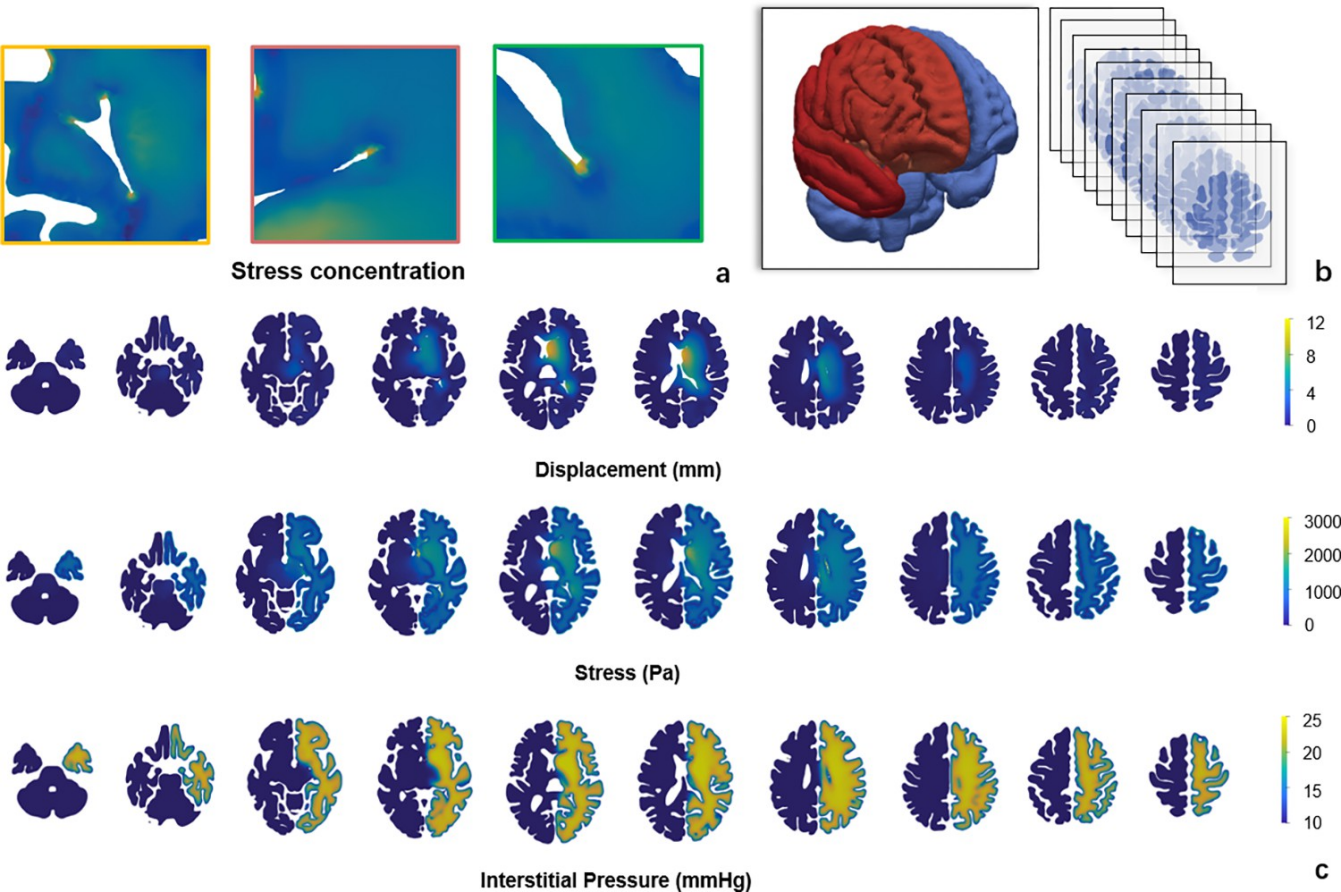
ICP, displacement and stress distribution in the brain (a) the stress concentration on the ventricle corners. (b) brain slices of the model, with red oedema geometry and blue whole brain geometry. (c) slices showing values of ICP, displacement and stress.

In brain oedema, there are multiple different types of damage, including stress damage and axonal damage caused by stretch. Therefore, in this section, we consider the distributions of ICP, displacement and tissue stress. In Fig 4, the ABP is 105 mmHg and the maximum ICP across the computational domain is found during the development of oedema. As the intracranial pressure rises, the oedema core swells and compresses the surrounding tissue, leading to a rise in tissue stress. The ICP rises in the entire hemisphere while the deformation occurs in certain regions on the ventricle surface. ICP value can be estimated by calculating the maximum pressure from the interstitial fluid compartment in the computational domain. It can be observed that stress concentrates at the “corners” of the ventricle. The periventricular stress concentration is much greater (approximately twice) than the maximum intraparenchymal stress.

### 2.3 ICP-MLS relationship

Due to the difficulties in measuring ICP in clinical settings, the MLS has been widely used to determine the severity of oedema. However, the ICP-MLS relationship has been shown to be multi-factorial and it is thus challenging to substitute MLS for accurate ICP estimation. Hence, to study the effects of these factors, i.e., patient-specific brain geometry, BBB damage severity and types of oedemas, are simulated to obtain ICP-MLS curves and to quantify the variability that can arise.

#### 2.3.1 Patient-specific geometry

**Fig 5:**
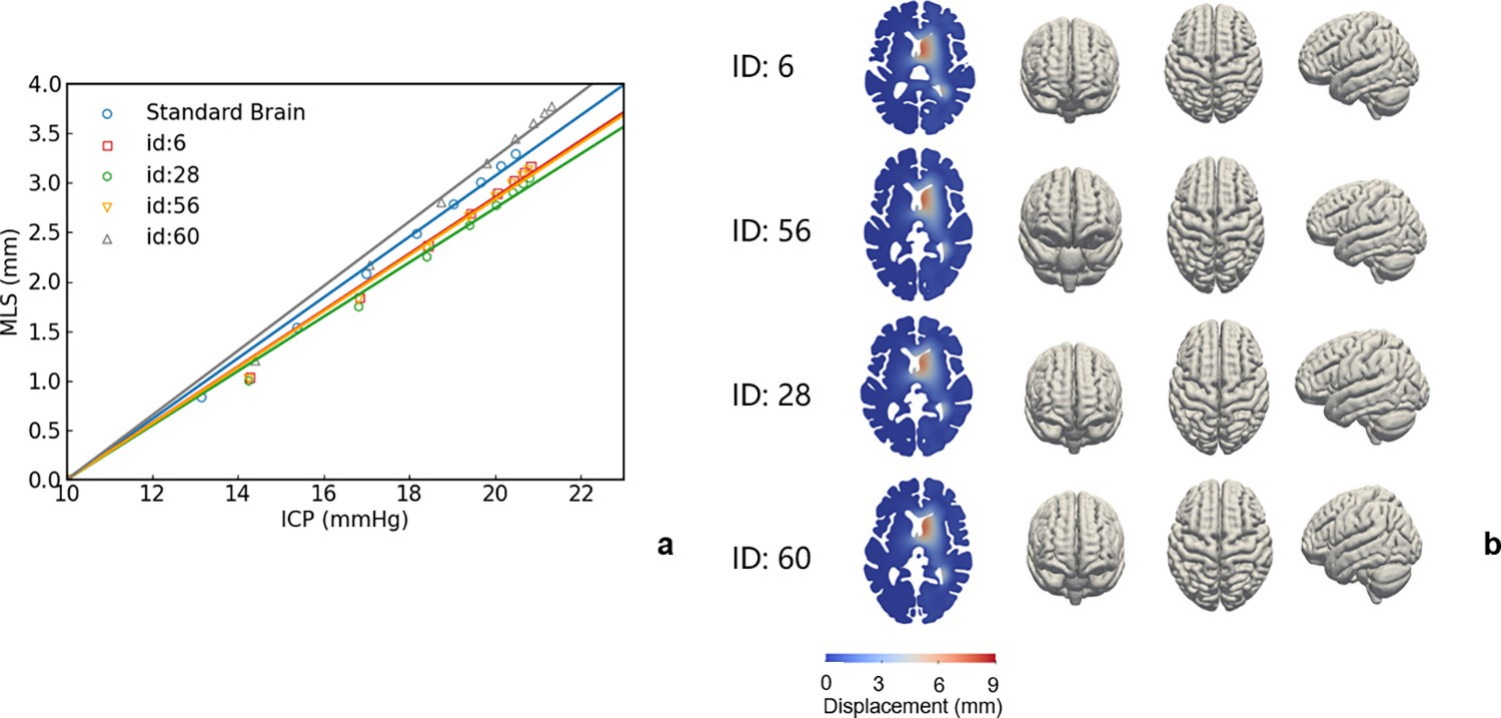
(a) ICP-MLS curves; and (b) patient-specific brain geometries obtained from affine transformation.

According to our previous study, the brain mesh is affine transformed according to CT imaging to obtain brain models that are similar to patient-specific brains [29]. Quasi-patient-specific geometries are obtained by first creating a general mesh template in the standard space and then an affine registration of this template to the tissue mask of each specific subject. This approach avoids time-consuming and potentially non-robust patient-specific geometry reconstruction. A mesh template is prepared from the averaged T1-weighted MNI template of the IXI555 cohort [38]. Here we study oedema in patient brains with hemispheric oedema and compare the ICP-MLS curves with the population-averaged brain (Fig 5). Linear fits are used to find the ICP-MLS slope and zero intercept is imposed in the linear fitting as the MLS is zero when there is no pressure rise in the healthy brain. In this study, 18 cases are simulated and the four with the most different ICP-MLS curves are shown in Fig 5. The oedema volumes, sagittal, coronal, and axial lengths of the patient brains are summarized in Table 1.

**Table 1:** Oedema volumes and the maximum lengths of the four brain geometries.

Patient	Volume of Oedema (ml)	Sagittal Length (cm)	Coronal Length (cm)	Axial Length (cm)
Standard Brain	55.75	13.8	17.4	15.5
6	42.62	13.0	15.7	14.6
28	45.18	12.6	16.9	14.7
56	42.42	12.7	16.2	14.8
60	59.59	14.1	17.4	17.0

**Fig 6:**
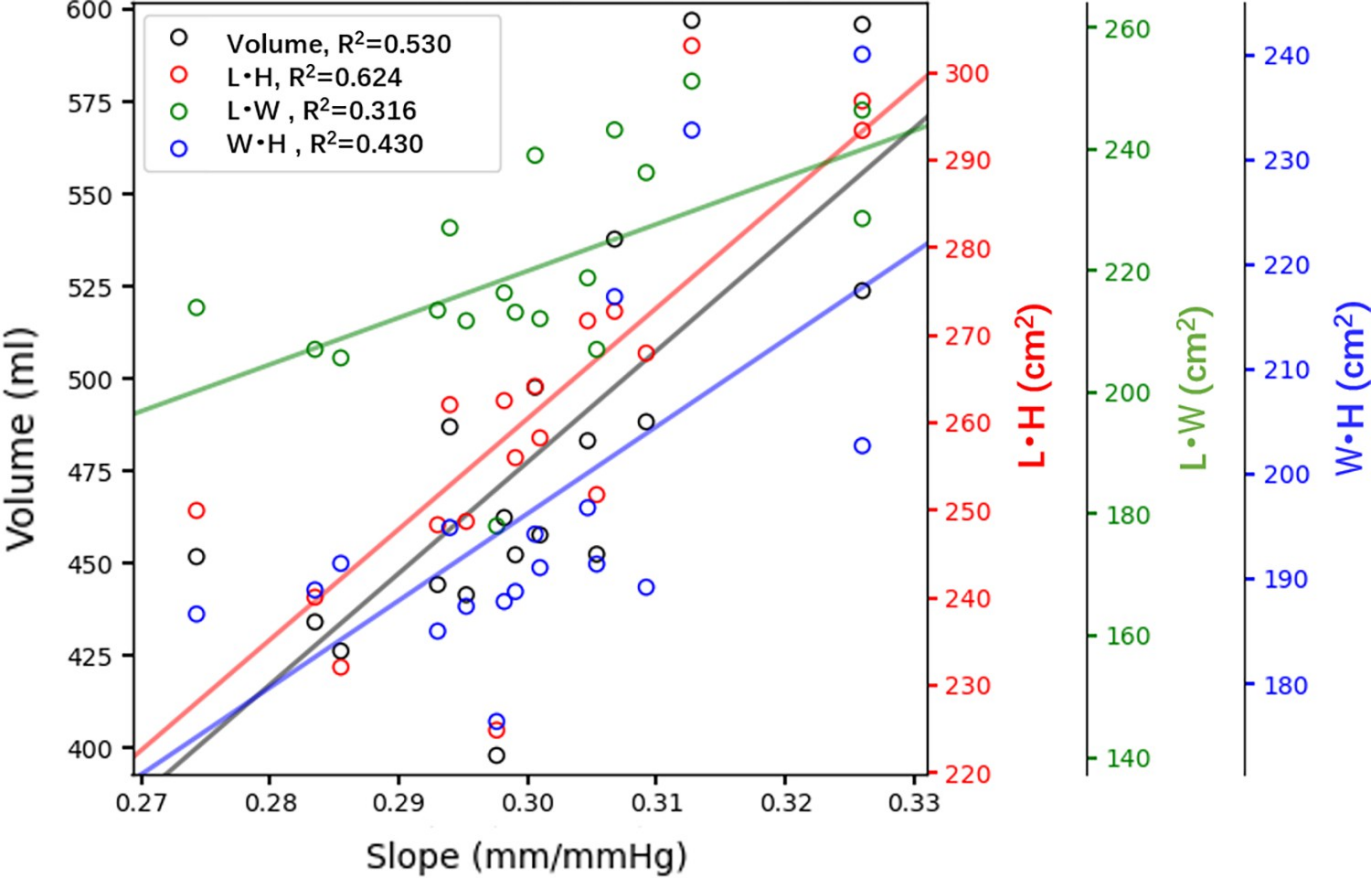
The factors that can affect the ICP-MLS relationship, where volume indicates the volume of oedema in different brains, and the L, W and H represent coronal, sagittal and axial lengths, respectively. The horizontal axis shows the slope of ICP-MLS curves, and the vertical axes show the geometrical features of the brains.

To illustrate the correlation between the ICP-MLS slope and geometrical features of the brain. The volume of oedema and 3 dimensions of the 18 patient-specific brains are plotted with the slopes of ICP-MLS curves (Fig 6). It can be seen that the ICP-MLS relationship is dependent upon the volume of the oedema, with an R^2^ of 0.530. Meanwhile, the plot of 3 dimensions shows the axial and coronal lengths of the patient brain correlate with the ICP-MLS slopes. In brains with larger axial and coronal lengths, the ventricle has a larger cross-section for brain tissue displacement from the right to left hemisphere and this indicates the ICP-MLS curve is affected by the geometry of the ventricle.

#### 2.3.2 Severity of BBB damage

**Fig 7:**
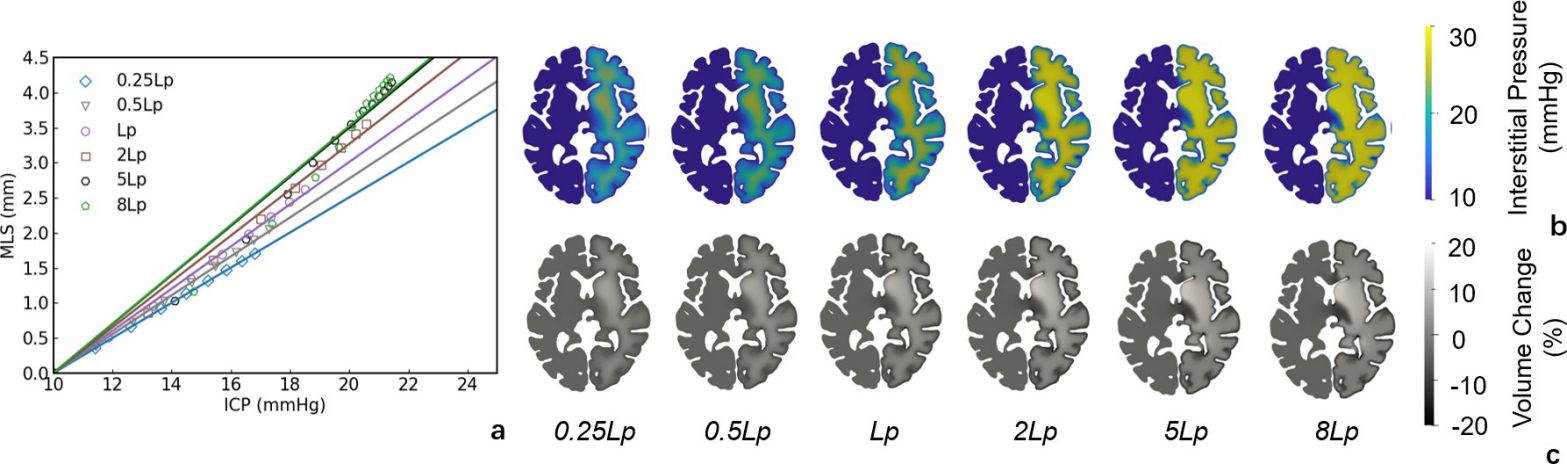
Effects of *L*_*p*_ value and the volume change in the oedema brain. The value of *L*_*p*_ rises from left to right. (a) ICP-MLS curves. (b) Interstitial pressure. (c) Volume changes.

Another factor that varies among patients is the severity of BBB damage. BBB damage depends on the effectiveness of the stroke therapy, age, medical history, collateral score, etc [39]. Due to the different extent of BBB damage, some patients present MLS whereas others do not. To investigate the effects of the severity of BBB damage, the *L*_*p*_ value is varied to analyse the ICP-MLS relations. As shown in Fig 7B, the ICP rise is distributed only in the oedema core when the *L*_*p*_ is small and the ICP becomes more evenly distributed across the hemisphere as *L*_*p*_ rises (representing more leaky blood vessels). Meanwhile, the volume change in the brain tissue can be defined as:

$$\Delta V=\frac{V_{c}-V_{i}}{V_{i}}\times 100\%$$ (1)

where the *V*_*c*_ is the volume of tissue after oedema occurrence and *V*_*i*_ is the tissue volume before oedema. As the BBB damage becomes more severe, the volume of the brain tissue also increases across a larger region, from the core in the oedema volume to almost the entire right hemisphere (Fig 7C). As the tissue swells, the increase in the volume in the oedema core compresses the surrounding tissue and leads to a tissue volume reduction (around 20%) at the periventricular region and at the pial surface. It is found that as *L*_*p*_ rises, the volume of fluid leakage into the interstitial space becomes greater and thus leads to larger MLS under the same maximum ICP rise (Fig 7A). However, when the increase of volume in the brain tissue almost spreads across the entire hemisphere (as shown in the 5*L*_*p*_ and 8*L*_*p*_ cases), the slope of the ICP-MLS curve stops increasing (Fig 7A).

#### 2.3.3 Types of oedema

**Fig 8:**
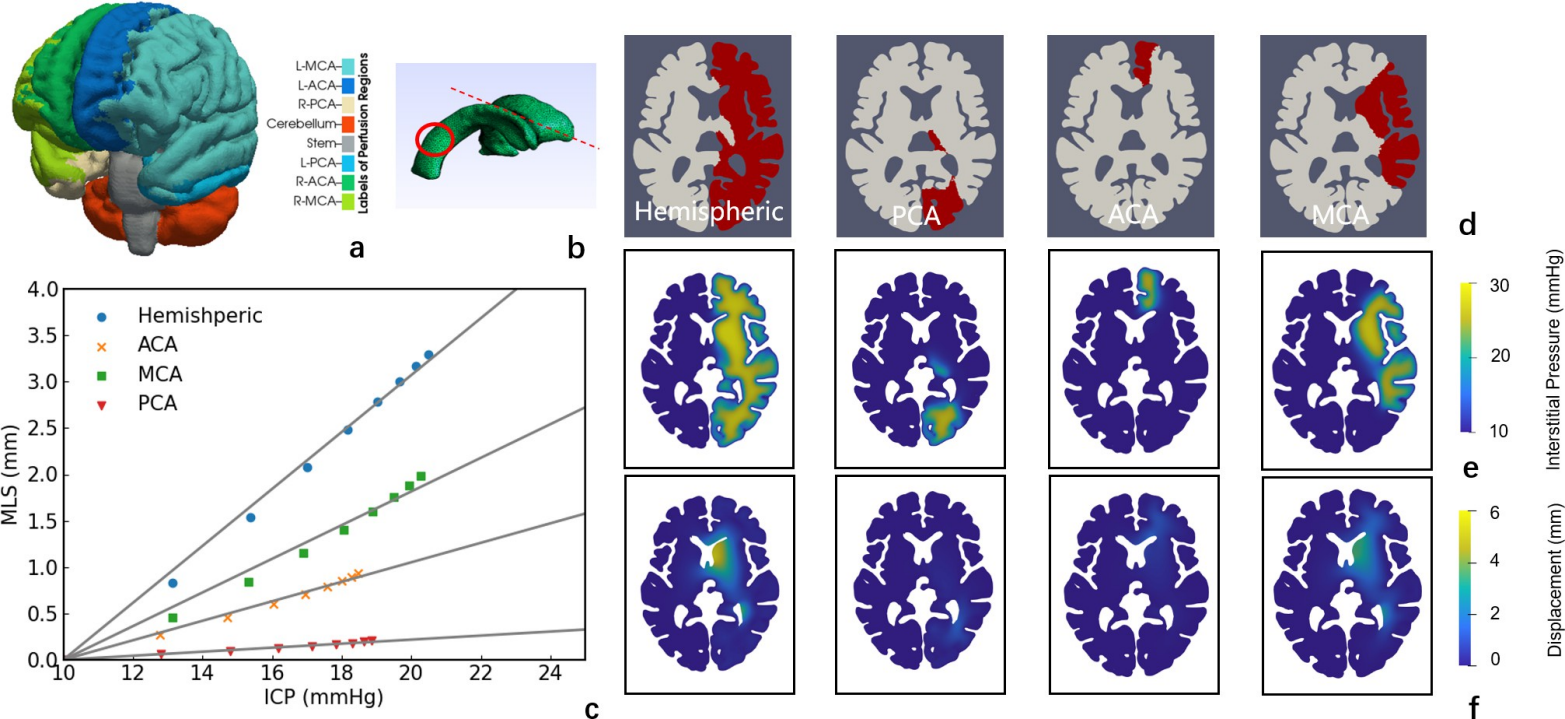
Simulations of different types of oedemas. (a) Subregions on brain model. (b) Ventricle geometry and midline of the brain. (c) MLS-ICP relations for different types of oedemas. (d) Hemispheric, PCA, ACA and MCA oedema geometries. (e) ICP rise. (f) Displacement.

Different types of stroke lead to different types of post-stroke oedema, including ACA, PCA, MCA, and hemispheric oedema, etc. MCA and hemispheric oedema are common types of oedema, whereas PCA and ACA are rare and are thus hard to investigate in clinical settings. In the model, oedema at different locations leads to differences in anatomical features and thus various ICP-MLS relations. According to a vascular territory atlas [40], each artery feeds one sub-territory at the pial surface, as shown in Fig 8A. In this section, different territories on the pial surface are occluded in the perfusion model to obtain damaged tissue volume for a range of oedema simulations.

The volumes of oedema cores are listed in Table 2. It can be noted that the ICP-MLS slopes of different types of oedemas vary significantly (Fig 8C and 8D). The pressure rise in the interstitial fluid compartment in different types of brain oedema is similar (Fig 8E). Meanwhile, the volume of the MCA oedema is twice the volume of PCA or ACA, while the volumes of PCA and ACA oedema are around the same. Although the PCA and ACA oedema are of similar volume, the MLS in ACA oedema reaches around 1 mm when ICP is 20 mmHg while the PCA oedema leads to almost no MLS. The PCA oedema, the displacement in the brain tissue concentrates at the right back of the ventricle and thus does not contribute to the MLS (Fig 8B and 8F). Meanwhile, the ACA oedema leads to much larger MLS as the swelling tissue compresses the tissue on the opposite side even though the deformation is restricted by the falx which is assumed to be rigid. The MLS caused by ACA can be larger if the falx is included as a part of the model. In addition, tissue swelling in MCA oedema pushes the brain from the side and therefore the MLS caused by MCA oedema is the largest of the three types of oedema.

**Table 2:** Volumes of different types of brain oedema.

Type of Oedema	Oedema Volume (ml)	Slope (mm/mmHg)
Hemispheric	53.78	0.307
PCA	7.00	0.022
ACA	8.52	0.105
MCA	16.98	0.181

## 3. Discussion

This paper proposes the first model for the study of post-stroke oedema with MLS under large deformation. Although computational models have been widely used for the study of brain oedema, post-stroke oedema has not been thoroughly investigated. Different from previous studies in which the oedema cores are assumed as growing volumes [30] or are assumed to occur in the entire brain [23,41], a perfusion component is utilised in our model for the simulation of different types of oedemas and to obtain the oedema volume. This provides more accurate population-averaged oedema locations and allows us to investigate the differences between different oedema types in more detail. Meanwhile, the population-averaged brain can be affine transformed to obtain patient-specific brains and to analyse the effects of their anatomical features. Furthermore, as the brain is modelled as a four-compartment poroelastic medium, it can thus provide overall information on blood pressure, interstitial pressure, displacement, stress and strain. This helps improve our understanding of post-stroke brain oedema as a multi-factorial process.

This model also incorporates for the first time the contact mechanics problem into the modelling of brain oedema. Previous studies assume the ventricle as a material with low shear modulus as an approximation of the fluidity of cerebrospinal fluid [30–32]. However, the compression of the ventricle mesh during the tissue swelling makes it challenging to simulate brain oedema with large deformations. In previous studies, most models only focus on marginal brain deformation and the MLS is only around 2 mm, which is smaller than the clinical threshold of severe oedema (5mm) [30–32]. In the model proposed here, the brain ventricle can collapse and therefore allows us to investigate brain oedema with MLS of around 7mm and to study severe oedema cases.

The simulation results are compared with the clinical data of 97 oedema patients for validation. The linear relationship between ABP and ICP observed in the patients is replicated in the model by varying arteriole blood pressure boundary conditions. The ICP values are chosen from different time points, ranging from 0 to 1000 s. The time taken to reach the ICP values in the clinical data are around 1000 second and this is much shorter than the time recorded (in days) in clinical settings. This is because the development of oedema is not just a fluid and solid mechanic process, and biochemical factors including oxidative stress, toxic substances and enzymes are involved in the damage of the BBB over a longer time period [42,43]. In clinical settings, the ICP can be measured both in the ventricle and in the parenchyma. As the gold standard of ICP measurement, the pressure in the ventricle is measured through an intraventricular catheter. However, the pressure represents the CSF pressure and is thus not able to reflect the pressure in the tissue. In this study, the intraparenchymal pressure is measured using the ICP micro transducer. Two outliers are shown in the boxplot (Fig 3) and the clinical data (range from 1 to 5.5 mm, with a mean value of 3.6 mm) are compared to the in-silico results (range from 0.5 to 6.3, with a mean value of 3.1 mm). Although there are limited clinical data available, this analysis provides some comparison between the clinical data and our computational model and helps determine the key parameters when using a poroelastic model for oedema modelling.

The ICP-MLS relationship is the most crucial for the estimation of brain oedema severity. Here, we only study hemispheric oedema in Figs 5 and 7. It is because the hemispheric oedema is the largest in volume and thus most sensitive to variations in geometry and *L*_*p*_. As shown in the results, the types of oedemas affect the linear relationship significantly (Fig 8) while the geometry and BBB damage severity can only lead to around 2 (Fig 5) and 5 (Fig 7) mmHg underestimation of ICP when MLS is 3.5 mm. Therefore, it is crucial to take the types of oedemas into consideration when estimating ICP from MLS. Especially, estimating ICP from MLS in PCA oedema leads to a very significant underestimation of ICP and thus should be considered in clinical studies. Furthermore, stress can restrict blood flow and lead to the rupture of blood vessels, with the compression/tension causing mechanical damage to axons. The simulation results show that under large deformation, the periventricular and intraparenchymal regions experience reduction and increase in tissue volume and the stress concentrates at the periventricular corners. This suggests that the periventricular regions are prone to vessel closure in oedema and therefore have a higher risk for secondary ischaemia and tissue damage. In the simulation results (as shown in Appendix B in S1 Text), both the ICP and MLS can predict intraparenchymal pressure well but do not predict the periventricular stress accurately. This indicates that the intraparenchymal stress is determined by the forces exerted by excessive ICP in the tissue, whereas the stress at the ventricle corners is affected by the geometry of brain and it is therefore less predictable and more challenging to evaluate its risks.

In the simulation results represented in the study, the impact of large deformation and contact on MLS is not obvious due to the relatively simple MLS measurement technique in the model. The MLS is simply measured as the maximum displacement on the midplane of the brain and the MLS shows a good linear relationship to ICP throughout this study. However, in the clinical measurements, the MLS is usually measured at brain structures that are more visible on imaging, (such as Foramen of Monro or a Bezier curve connecting posterior and anterior brain falx) [14] and the locations of MLS measurement can depend on the protocols and algorithms employed. Here, we summarised the ICP-MLS relationship by probing the MLS at different locations on the ventricle surface, as shown in Fig B in S1 Text. The results show a significant variation in the ICP-MLS slopes and different patterns under different deformation before and after contact (Fig B in S1 Text). While point 1, 2 and 3 show a reduction in ICP-MLS slope, point 4 shows a slight rise in the ICP-MLS slope. This is probably because of the additional obstruction induced by contact and therefore additional deformation concentration at certain locations. This indicates that the measurement of MLS can be sensitive to the location of measurements, and this will require more detailed investigation by using algorithms to mimic the realistic MLS measurements in our model. Different from the physiological aspects (brain geometry, oedema type, BBB damage), this constitutes another important aspect that can induce error in ICP-MLS estimation as it arises from the limitations of measurement techniques and protocols.

### Limitations

It should be noted that there are several limitations to this study. Firstly, many parameters have been used in the multicompartment model. Some of them are challenging to measure in clinical settings or experiments. For example, the fluid filtration of the capillary wallhas been measured in frog models [44] but there is still a lack of data on the *L*_*p*_ values in the human brain [45]. Meanwhile, some parameters such as the Poisson ratio and shear modulus of the brain tissue remain controversial. The values of the mechanical property parameters are affected by tissue sampling and experimental techniques and thus vary dramatically in previous literature.

Secondly, due to the danger and high risk of intraparenchymal pressure measurement, the data on ICP are very limited. In this study, we use the data from 97 patients whose oedema was caused by traumatic brain injury (TBI) for the validation of our model. Although both TBI and post-stroke oedema are caused by BBB damage [46,47], the TBI oedema takes longer to develop, whereas post-stroke oedema can occur in hours or days and the difference between the mechanisms is still unclear. Thirdly, the deformation of the brain can be more complicated because of the realistic boundary conditions. In the model, the pial surface of the brain imposes a zero-displacement boundary condition and the falx is not included in the model and is thus considered non-deformable.

Finally, large blood vessels can play a role in ischaemic stroke and thus affect the oedema development, as shown in previous work [48,49]. The large deformation can lead to vessel distortion and thus a more complicated coupling behaviour between blood flow and tissue deformation. Since there is still a lack of mathematical description of such coupling behaviour, large vessels have not been included in the model.

In conclusion, this is the first model studying brain swelling and ventricle collapse in brain oedema. The simulation results are found to be in good agreement with the clinical data of 97 patients. Stress, volumetric change, interstitial pressure, blood pressure, compression/tension and MLS of the oedema brain are presented and the stress concentration in the periventricular region is highlighted. Furthermore, various factors (patient brain geometry, BBB damage severity and oedema types) that can affect the estimation of ICP from MLS are investigated to assist clinical decision-making. This model can hopefully be further developed to achieve better accuracy and to provide more detailed guidelines for the clinical treatment of oedema.

## 4. Methods

### Ethics statement

The experimental protocol and informed consent were approved by the Institutional Review Board (Protocol 30 REC 1997/290, Addenbrooke’s Hospital, University of Cambridge, UK). For patients monitored before 1997, the Neurocritical Care Users’ Committee allowed TCD for the assessment of TBI patients. Informed written consent was obtained from all individual participants included in the study. Further use of the anonymised data was allowed as a part of clinical audits.

The modelling pipeline consists of a perfusion component and an oedema component for the simulation of healthy blood perfusion, stroke blood perfusion, and oedema, respectively. The healthy and stroke blood perfusion simulated in the perfusion model is utilized to obtain the oedema core, which will then be imported into the oedema model for oedema simulation. The models are presented individually before being coupled together.

### 4.1 Perfusion model

#### 4.1.1 Governing equations

In the perfusion component, the brain is modelled as a three-compartment porous medium including arteriole, venule, and capillary blood compartments [26] for the simulation of blood flow in healthy and stroke scenarios. The governing equations have also been utilised for the simulation of osmotherapy [33] and the investigation of haemorrhagic transformation [34] in our previous studies. The governing equations describing three porous compartments are taken directly from these previous studies:

$$\nabla ∙(K_{a}\nabla p_{a}){{=-\omega }_{ac}∙(p}_{a}-p_{c}),$$ (2)

$$\nabla ∙(K_{c}\nabla p_{c}){{=\omega }_{ac}∙(p}_{a}-p_{c})-{\omega_{cv}∙(p}_{c}-p_{v}),$$ (3)

$$\nabla ∙(K_{v}\nabla p_{v}){{=\omega }_{cv}∙(p}_{c}-p_{v}),$$ (4)

where *p*_*a*_, *p*_*c*_ and *p*_*v*_ are the Darcy pressures in the arteriole, capillary, and venule compartments respectively. ***K***_***i***_ is the permeability tensor in compartment *i*, whereas *ω*_*ij*_ represents the fluid transfer coefficient between compartments *i* and *j*. In the equations, the LHS terms are the blood flow within each compartment and thus represent the blood flow within vessel networks (arteriole, capillary, and venule). Meanwhile, the RHS terms represent the blood flow between compartments and describe the blood flow between arteriole, capillary, and venule vessels. In our previous studies [26,33], the arterioles and venules are penetrating vessels perpendicular to the pial surface. Arteriole and venule compartments are thus modelled as anisotropic and inhomogeneous porous media, whereas the capillary vessels form an interconnected network, and this compartment is therefore considered a porous medium with isotropic and homogeneous permeability. In Eq 3, the ***K***_***c***_ tensor field is replaced with a *K*_*c*_ scalar field.

#### 4.1.2 Boundary conditions

Blood is supplied to the brain through arterioles to feed the capillary network compartment and returns to the veins through venules at the pial surface. This means there is blood flow through the pial surface in the arteriole and venule compartments while the blood flux through other surfaces is zero. According to a vascular territory atlas [40], each artery supplies one sub-territory at the pial surface. In the model proposed here, the blood pressure in the venule compartment on the cortical surface is assumed to be 15 mmHg, while the pressure in the arterioles at the pial surface is around 90 mmHg. In the case of artery occlusion, blood flow through the perfusion territory of the occluded vessel is set to zero, but pressure on the pial surface is assumed to remain constant in other regions. The boundary conditions of the perfusion model are summarized in Table 3.

**Table 3:** Boundary conditions for the perfusion model.

	Cortical Surface	Ventricle Surface
**Arteriole Blood**	$p_{a}=90mmHg(non-occluded\;regions)$; ${\nabla K_{a}p}_{a}∙\overset{\to }{n}=0(occluded\;region)$;	${\nabla K_{a}p}_{a}∙\overset{\to }{n}=0$
**Capillary Blood**	${K_{c}\nabla p}_{c}∙\overset{\to }{n}=0$	${K_{c}\nabla p}_{c}∙\overset{\to }{n}=0$
**Venule Blood**	*p*_*v*_ = 15mmHg	${\nabla K_{v}p}_{v}∙\overset{\to }{n}=0$

### 4.2 Oedema model

#### 4.2.1 Governing equations

Because of the relatively slow rate of interstitial space fluid flow compared to blood flow, the flow exchange between the capillary network and the interstitial space is negligible in a healthy brain. Therefore, only three compartments are used in the perfusion model. In the oedema model, however, the brain is damaged and additional fluid leaks into the interstitial space and leads to brain oedema. Therefore, additional compartments for interstitial fluid and brain deformation are required for the simulation of ICP and MLS in a damaged brain. In the oedema model, the brain is assumed to be a poroelastic medium consisting of five compartments, i.e., tissue, interstitial space, arteriole, capillary, and venule network. The maximum pressure in the interstitial compartment is utilised to define ICP. Meanwhile, a threshold of 70% or greater reduction in blood perfusion has been widely used in clinical settings [50, 51, 52] and therefore is employed to here define the location of leaking vessels in the model. The corresponding governing equations are given as:

$$c_{w}\frac{\partial p_{w}}{\partial t}=\nabla ∙(K_{w}\nabla p_{w})+S_{cw},$$ (5)

$$c_{b}\frac{\partial p_{a}}{\partial t}=\nabla ∙(K_{a}\nabla p_{a}){{-\omega }_{ac}∙(p}_{a}-p_{c}),$$ (6)

$$c_{b}\frac{\partial p_{c}}{\partial t}=\nabla ∙(K_{c}\nabla p_{c}){{+\omega }_{ac}∙(p}_{a}-p_{c})-{\omega_{cv}∙(p}_{c}-p_{v})-S_{cw},$$ (7)

$$c_{b}\frac{\partial p_{v}}{\partial t}=\nabla ∙(K_{v}\nabla p_{v}){{+\omega }_{cv}∙(p}_{c}-p_{v}),$$ (8)

$$G\nabla^{2}\overset{\to }{u}+(G+\lambda )\nabla \varepsilon =\nabla p_{w},$$ (9)

where *c*_*w*_ and *c*_*b*_ are the storage factors of the interstitial fluid and blood in the brain tissue. The parameters *p*_*w*_ and *K*_*w*_ are the pressure and permeability of the interstitial fluid space. *ε* is the dilatational strain. *G* and *λ* are the shear modulus and Poisson ratio. The term*S*_*cw*_ represents the fluid transport from the vasculature into the interstitial space. It should be noted that here we neglected the time derivative of displacement in the poroelastic theory (Eq 5) [23,53], and the poroelastic problem can thus be solved in a decoupled manner. This is because brain oedema develops over a long time scale and the process is highly dependent on the breakdown of the BBB. This makes the pressure a slow time scale (days or weeks) [42,43] variant whilst the deformation is at a much faster time scale (seconds or minutes) [54]. In oedema modelling, time-scale separation can be used and the displacement can always reach equilibrium. Therefore, the time derivative of displacement term becomes negligible.

The value of the *S*_*cw*_ term in the normal BBB has been estimated by Fraser et al. [44] and this value can increase by more than 100 times the normal value [55]. Due to the large difference between the BBB permeability in the damaged region and the healthy region, the flow from capillary blood to the interstitial space in healthy tissue is thus neglected. Meanwhile, the leakage of fluid into the interstitial space *S*_*cw*_ can be derived from modified Starling’s principle and Donnan’s Effect [56–58]:

$$S_{cw}=\left\{ \begin{array}{c} 2n_{b}\frac{L_{p}}{R_{c}}\left[{(p}_{c}-p_{w})-\sigma \Pi_{c}\right],Oedema\\ 0,Healthytissue \end{array}\right.$$ (10)

The term representing fluid transfer between the capillary and the interstitial fluid compartments (*S*_*cw*_) is derived in Appendix A in S1 Text. *L*_*p*_ is the hydraulic permeability of the capillary wall, *Π*_*c*_ is the osmotic pressure for the plasma components in the blood, *σ* is the reflection coefficient of the original plasma composition, *n*_*b*_ is the volume fraction of blood vessels in a unit volume of brain tissue, and *R*_*c*_ is the mean vessel radius.

#### 4.2.2 Boundary conditions

In the oedema model, the blood perfusion is restored and thus the arteriole pressure at the pial surface is constant. Therefore, the boundary conditions of the blood compartments are the same as the healthy blood perfusion listed in Table 4. For the interstitial compartment, the CSF in the ventricle and the subarachnoid space are compliable and the pressure in the CSF space is thus considered constant. The brain tissue is connected to the skull through three layers: dura mater, arachnoid and pia mater, and their stiffness is generally much greater than the brain tissue and can thus be considered rigid [23]. Therefore, the displacement at the pial surface is assumed to be negligible and a fixed boundary condition is thus imposed. As to the ventricle surface, however, the CSF in the ventricle does not resist any displacement and the stress on the ventricle surface is thus balanced by the CSF pressure in the ventricle. Meanwhile the ICP and venule pressure values are chosen to be similar as the venule pressure follows the ICP in clinical settings.

**Table 4:** Boundary conditions for the oedema model.

	Cortical Surface	Ventricle Surface
**Arteriole Blood**	*p*_*a*_ = 90mmHg	${\nabla K_{a}p}_{a}∙\overset{\to }{n}=0$
**Capillary Blood**	${K_{c}\nabla p}_{c}∙\overset{\to }{n}=0$	${K_{c}\nabla p}_{c}∙\overset{\to }{n}=0$
**Venule Blood**	*p*_*v*_ = 15mmHg	${\nabla K_{v}p}_{v}∙\overset{\to }{n}=0$
**Interstitial Space**	*p*_*w*_ = 10mmHg	*p*_*w*_ = 10mmHg
**Tissue**	$\overset{\to }{u}=\overset{\to }{0}$	$\sigma ∙\overset{\to }{n}=\overset{\to }{0}$

### 4.3. Parameters

The parameter values used here have been presented in our previous studies and were also thoroughly investigated in previous studies by other authors. The parameter values utilised in the modelling presented here are listed in Table 5.

**Table 5:** Values of model parameters used in the perfusion, oedema and osmotherapy models and their sources.

Parameter	Value	Reference(s)	Parameter	Value	Reference(s)
*c*_*b*_	1.59×10^−3^ Pa^-1^	[59]	*p*_*v*_ at cortical surface	2000 Pa = 15 mmHg	[26]
*c*_*w*_	3.08×10^−4^ Pa^-1^	Derived from [60, 61, 62]	*R*_*c*_	5×10^−6^ m	[63]
*G*	592.7 Pa	[54]	*σ*	0.65	Estimated from the initial ICP in oedema
***K***_***a***_	1.234 mm^3^s kg^−1^	Estimated by 0-order arteriole diameters and vessel density [26]	*p*_*a*_ at cortical surface	12000 Pa = 90 mmHg	[26]
*K*_*c*_	4.28×10^−3^ mm^3^s kg^−1^	(1.0×10^−15^ m^2^, [64]) (1.0×10^−10^ m^2^, [23])	*Π*_*c*_	2445 Pa	[59]
***K***_***v***_	2.468 mm^3^s kg^−1^	Estimated by pressure and blood perfusion rate [26]	*ω*_*ac*_	1.326×10^−6^Pa^−1^s^−1^	Derived from perfusion in grey and white matter [26, 27]
*K*_*w*_	3.6×10^−3^ mm^3^s kg^−1^	[59]	*ω*_*cv*_	4.641×10^−6^Pa^−1^s^−1^	
*L*_*p*_	3.0×10^−11^ m/s.Pa	[59]	*ν*	0.35	[56]
*n*_*b*_	0.03	[65]			

### 4.4 Numerical method

The governing equations are solved numerically using Python with a high-performance open-source finite element library, FEniCS [66, 67]. The highly coupled equations in the Finite Element model are solved in a mixed function space describing the multi-compartment dynamic system. The contact problem is solved using FEniCS, with a self-developed Augmented Lagrangian method to solve the self-contact of the brain ventricle during oedema. The method has been widely used for simulation in previous investigations of biomechanical problems [68, 69]. The equations implemented to solve the contact mechanics are:

$$G(u,v)+\int_{\Gamma }(\lambda_{N}^{\left(k\right)}+\varepsilon_{N}g)v∙n∙d\Gamma$$ (11)

$$\lambda_{N}^{\left(k+1\right)}=\langle \lambda_{N}^{\left(k\right)}+\varepsilon_{N}g\rangle$$ (12)

where 〈∙〉 is the Macaulay bracket, *g* is the penetration depth after brain deformation, ***n*** is the contact surface normal vector. ***u*** and ***v*** denote the displacement and the variation of displacement, respectively. *G*(***u*, *v***) is the potential energy of non-contact terms. *λ*_*N*_ is the Lagrange multiplier and *ε*_*N*_ is the penalty coefficient and they are used to impose a contact pressure on the contact boundary to achieve convergence and to avoid large penetration. A large penalty coefficient can lead to divergence, whereas a small penalty makes it time-consuming to achieve convergence. Therefore, different penalty coefficients are chosen for different simulation cases.

**Fig 9:**
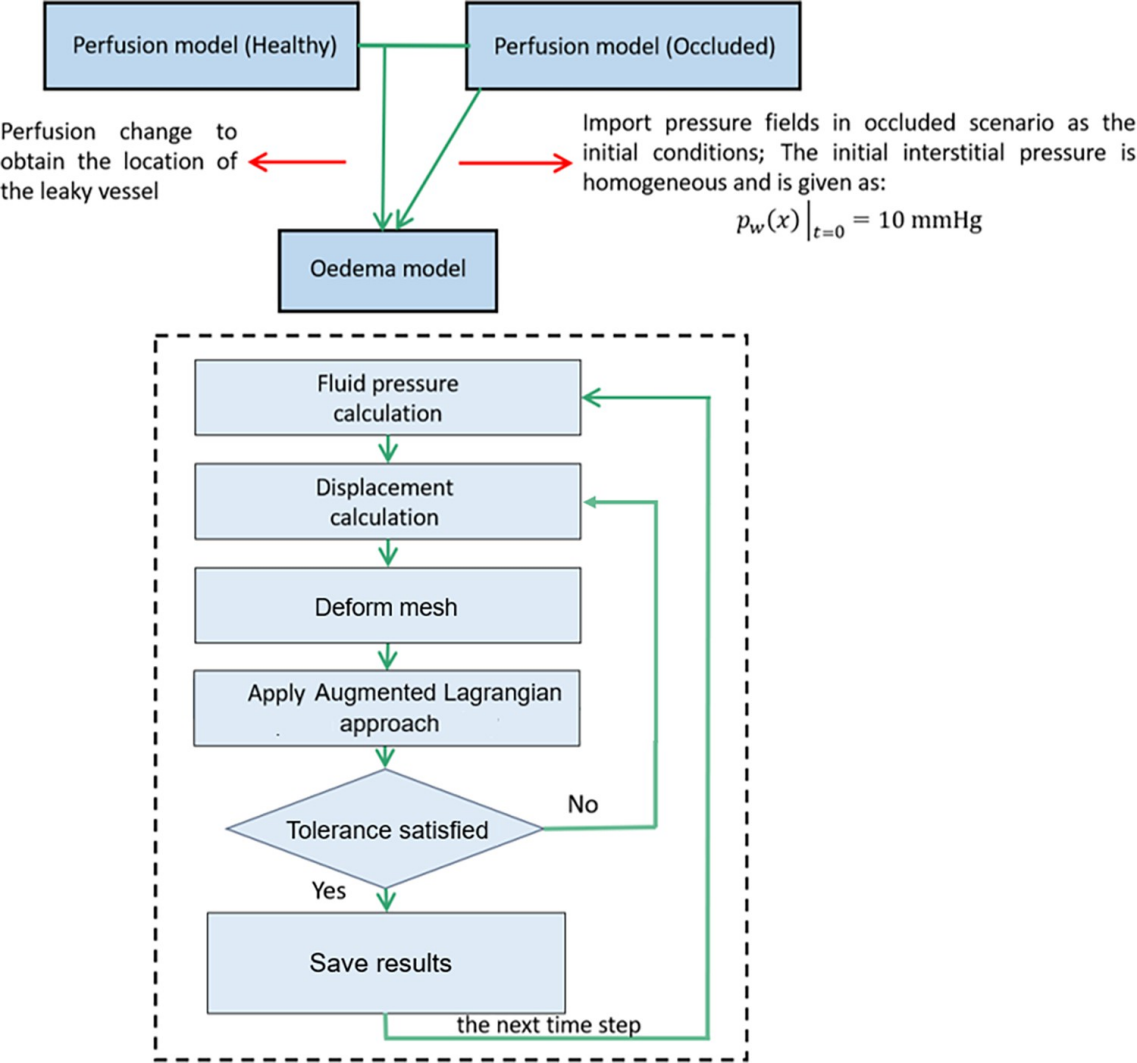
Flowchart of the computation algorithm for mesh deformation.

The iterations and simulation algorithms are shown in the flowchart depicted in Fig 9. The time steps are discretized using a backward Euler scheme throughout the model. The pressure field is first computed and then utilized to compute the displacement. The rise in ICP leads to tissue deformation and thus potential contact between ventricle surfaces. The collision between mesh elements is detected using BoundingBoxTree toolset in FEniCS after each iteration and the penetration depth of each penetrating mesh nodes is measured to obtain *g* and to generate the *λ*_*N*_ function. We use SciPy [70] and PETSc toolkits to assemble and solve stiffness matrices and residual forces generated from FEniCS. As the ventricle collapses, the surface of the ventricle contacts and the Lagrange multiplier is augmented to avoid large penetration of brain tissue. Once the Lagrange multiplier is found to keep penetration within tolerance, the results are saved and used for the simulation in the next time step. Details of the implementation of the contact algorithm and convergence criteria are introduced in Appendix D in S1 Text.

The pressure in each compartment (*p*_*i*_) is discretised with piecewise linear Lagrange (CG) elements. Meanwhile, the permeabilities (***K***_***i***_) and the other coefficients (*ω*_*ij*_, *L*_*p*_, etc) used in the model are represented in piecewise constant (dP0) scalar function spaces, respectively. The pressure field computed from the previous step is then used for the simulation of the displacement field, where the displacement is discretised with the Continuous Galerkin (CG) Method. The flow fields, the blood perfusion and the displacement are all computed in P1 elements and are projected using the BiCojungate Gradient STABilised method (BiCGSTAB) with an Algebraic MultiGrid (AMG) preconditioner.

### 4.5 Clinical data

The dataset used here is obtained from a retrospective cohort of 97 oedema patients who had traumatic brain injury (TBI) and were admitted to the Neurosciences Critical Care Unit between 1995–2002 in Addenbrooke’s Hospital, University of Cambridge, UK. Data were recorded and re-analysed anonymously under ethical protocol 30 REC 97/291. The median age was 34 years (range 13–76 years; 78% males). ABP was measured directly from the radial artery levelled at the level of the heart (Baxter Health Care Corp.). ICP was monitored continuously using a microtransducer (Direct Pressure Monitor; Camino Laboratories, or MicroSensors ICP Transducer; Codman and Shurtleff, Inc.), inserted intraparenchymally into the right frontal region. Among them, 24 presented unilateral lesions and midline shifts were confirmed by CT imaging [71]. The midline shift was measured in millimetres according to the scale on the CT scan. We assigned a positive value to the displacement of the midline from the right to the left side, and a negative value to displacement of the midline from the left to the right side. No midline shift was graded as a zero value. The median number of daily recordings was 3 days (range 1–11 days). As the post-ischaemic stroke is usually not as severe as the TBI, data on the intraparenchymal monitoring of ICP is very rare. Therefore, the TBI data are utilised in this in-silico study to obtain proper mechanical properties, including the shear modulus and Poisson ratio of the brain tissue.

## Supporting information

S1 Text **Fig A in S1 Text.** The intraparenchymal stress and periventricular stress vs ICP and MLS, where the blue and red lines are the best linear fit of the stress curves. (a) ICP-intraparenchymal stress. (b) MLS-intraparenchymal stress. (c) ICP-periventricular stress. (d) MLS-periventricular stress. **Fig B in S1 Text.** The locations of MLS probing and the ICP-MLS curves are marked with brown, blue, green and orange for point 1, 2, 3 and 4, respectively. The red lines show the time when contact is detected, and the grey doted lines show the linear fit of MLS data before contact. (a) periventricular points. (b) Point 1 ICP-MLS curve. (c) Point 2 ICP-MLS curve. (d) Point 3 ICP-MLS curve. (e) Point 4 ICP-MLS curve.
(DOCX)
