# Supplementary material for: Modelling midline shift and ventricle collapse in cerebral oedema following acute ischaemic stroke
Source: PLoS Comput Biol. 2024 May 28;20(5):e1012145. doi: 10.1371/journal.pcbi.1012145 (PMC11161059; doi:10.1371/journal.pcbi.1012145)
Supplement: S1 Text — Fig A in S1 Text. The intraparenchymal stress and periventricular stress vs ICP and MLS, where the blue and red lines are the best linear fit of the stress curves. (a) ICP-intraparenchymal stress. (b) MLS-intraparenchymal stress. (c) ICP-periventricular stress. (d) MLS-periventricular stress. Fig B in S1 Text. The locations of MLS probing and the ICP-MLS curves are marked with brown, blue, green and orange for point 1, 2, 3 and 4, respectively. The red lines show the time when contact is detected, and the grey doted lines show the linear fit of MLS data before contact. (a) periventricular points. (b) Point 1 ICP-MLS curve. (c) Point 2 ICP-MLS curve. (d) Point 3 ICP-MLS curve. (e) Point 4 ICP-MLS curve. (DOCX) [file pcbi.1012145.s001.docx]

**Appendix A: Derivation of Fluid Filtration through Capillary Wall**

Here, we employ the filtration theory and the Donnan effect, which has been used to model brain tissue in previous studies [1], to derive $S_{cw} ,$ i.e., fluid filtration through the damaged BBB. The filtration flux, $J_{VA}$, can be described by the following filtration equation as shown below.

$J_{VA}= L_{p}\left[ {(p}_{c}-p_{w})-\sum_{m=1}^{M} {\sigma(\Pi_{c}-\Pi_{w})}_{m} \right]$ , (A1)

where $L_{p}$ is the hydraulic permeability of the capillary wall, $m$ is the different solutes, $p_{i}$ is the hydrostatic pressure with subscripts that represent capillary blood pressure and interstitial fluid pressure. Meanwhile, $\Pi_{c}$ is the osmotic pressure for each water-soluble solute present in the blood plasma and interstitial fluid. $\sigma$ is the reflection coefficient that varies from 0 to 1, where a value of 1 means that no solute can flow through the BBB. Meanwhile, the difference in osmotic pressure can be described by the equation:

$\Pi_{c}-\Pi_{w}=\sigma\Pi_{c}\frac{1-exp(Pe)}{1-\sigma\cdot exp(Pe)}$, (A2)

where $\mathrm{Pe}$ is the $P\acute{e}\mathrm{clet}$ number. Here the $P\acute{e}\mathrm{clet}$ number is much smaller than one [2] and for a fixed composition of the filtration model, we thus have:

$J_{VA}= L_{p}\left[ {(P}_{c}-P_{w})-\sigma\Pi_{c} \right]$, (A3)

Meanwhile, the fluid transfer between capillary network and interstitial space $S_{cw}$ can be given as

$S_{cw}=J_{VA}\frac{n_{b}}{\pi{R_{c}}^{2}}\int_{0}^{\infty} p(c)dc$, (A4)

where $n_{b}$ is the volume fraction of blood vessel in a unit volume of brain tissue, $R_{c}$ is vessel radius and $p(c)$, at each point in space over the distribution of vessel circumferences. By assuming that the perimeter of blood vessels remains constant, we have:

$\int_{0}^{\infty} p(c)dc=2\pi R_{c}$, (A5)

Substituting Equations A3 and A5 into Equation A4, $S_{cw}$ can therefore be written as:

$S_{cw}= 2n_{b}\frac{L_{p}}{R_{c}}\left[ {(P}_{c}-P_{w})-\sigma\Pi_{c} \right]$. (A6)

**Appendix B: Intraparenchymal and Periventricular Stress in Oedema Brains in Quasi-patient-specific Brains**

The stress in the parenchyma and at the ventricle corners can be plotted against the ICP and MLS, respectively for 18 patients’ brains. As shown in the figure, the intraparenchymal stress is more related to the deformation of the brain and ICP values, whereas the periventricular stress is not as predictable as intraparenchymal stress. This indicates that the intraparenchymal stress is determined by the forces exerted by excessive ICP in the tissue and the periventricular stress is determined by the local tissue deformation and quasi patient-specific geometries of the brains.


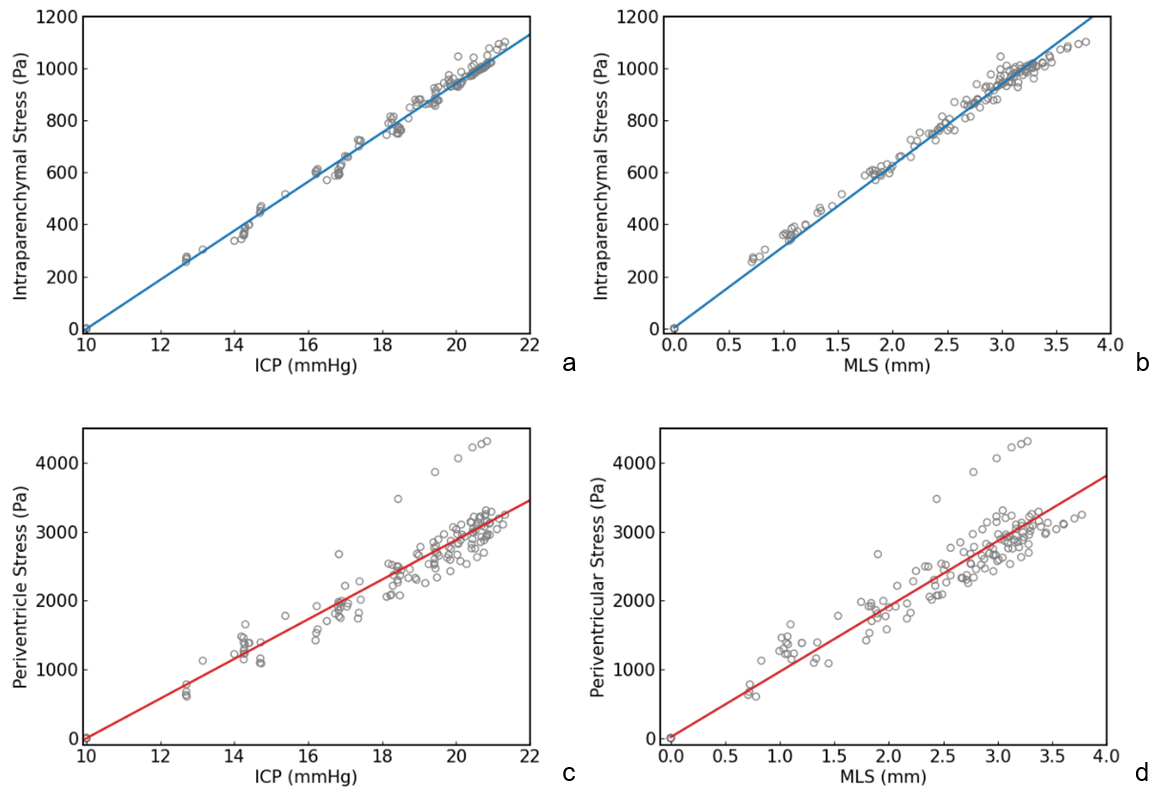


**Fig A.** The intraparenchymal stress and periventricular stress vs ICP and MLS, where the blue and red lines are the best linear fit of the stress curves. (a) ICP-intraparenchymal stress. (b) MLS-intraparenchymal stress. (c) ICP-periventricular stress. (d) MLS-periventricular stress.

**Appendix C: Probing MLS at Different Locations**

Here, we show the MLS can be sensitive to the locations of measurement by probing MLS at different periventricular points, as shown in the figure:


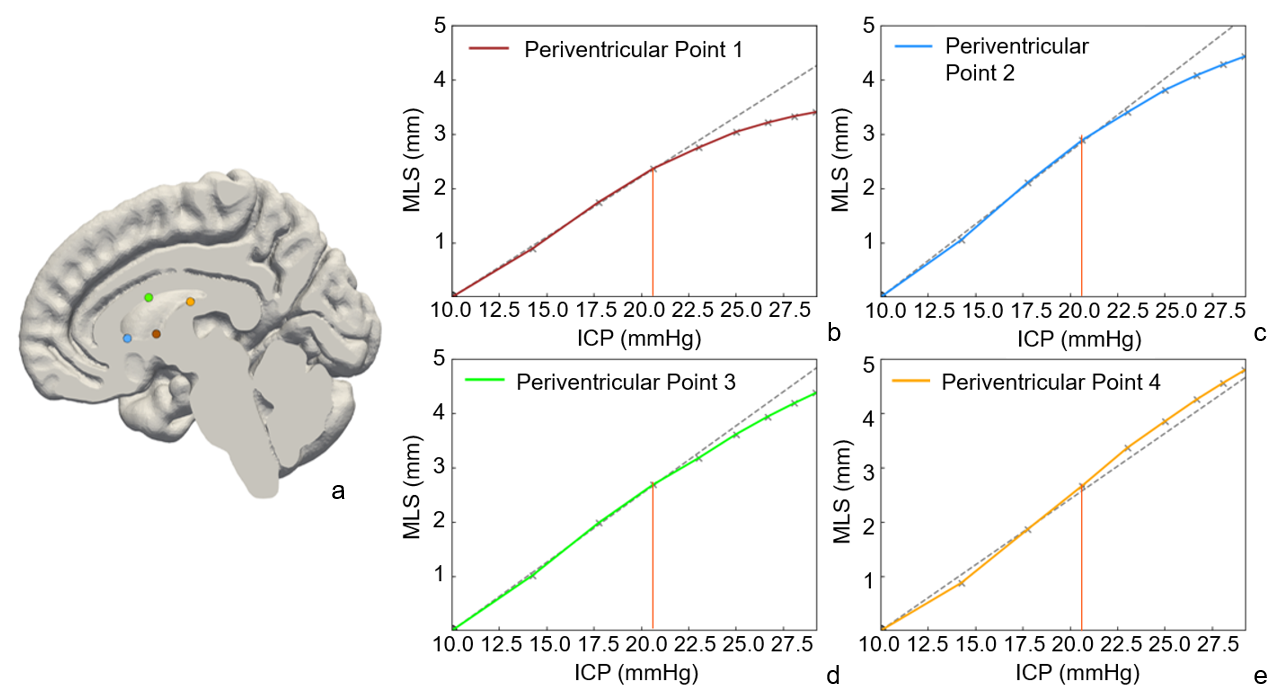


**Fig B.** The locations of MLS probing and the ICP-MLS curves are marked with brown, blue, green and orange for point 1, 2, 3 and 4, respectively. The red lines show the time when contact is detected, and the grey doted lines show the linear fit of MLS data before contact. (a) periventricular points. (b) Point 1 ICP-MLS curve. (c) Point 2 ICP-MLS curve. (d) Point 3 ICP-MLS curve. (e) Point 4 ICP-MLS curve.

s

As shown in the figure, the MLS at the final step can vary from around 3 to 5 mm for point 1 and point 4. Meanwhile, a significant difference in the ICP-MLS slope can be found at point 1 and point 2. This indicates that the MLS measurement can be sensitive to the location and protocol of MLS measurements.

**Appendix D: Mechanical Solver for Contact Mechanics**

In the implementation of contact mechanics algorithm, we use Laursen’s contact formulation [3], where the displacement and the Lagrange multiplier are solved until convergence in both the inner and outer loop. For simplicity, we do not use the BFGS solver in the original method [3] and we replace the line search with simpler relaxation coefficients. The algorithm can be written as:

| **Contact Mechanics Solver** |
| --- |
| 1. **Initialise the iteration process** |
| 1. Set initial values of displacement $\boldsymbol{u}$**_0_** and $\lambda$^(0)^ |
| 1. Compute initial search direction $\Delta\boldsymbol{u}$**_0_** and compute initial energy $G_{I}=\Delta\boldsymbol{u}_{\boldsymbol{0}}*R$($\boldsymbol{u}_{\boldsymbol{0}}; \lambda$^(0^), where |
| $R$($\boldsymbol{u}_{\boldsymbol{0}}; \lambda$^(0^) is the residual force |
| 1. Set iteration counters, $i=0, k=0$ |
| 1. **Loop on displacement until equilibrium** |
| 1. Relaxation of displacement and move mesh |
| 1. $i +=1$ |
| 1. Solve $\Delta\boldsymbol{u}_{\boldsymbol{i}}$ and compute the energy of current step $G$_c_ |
| 1. If ($G_{c}$ < ETOL${*G}_{I}$), Then GOTO step 3 |
| 1. **Augment** |
| 1. Compute new Lagrange multiplier $\lambda^{tr}$ |
| 1. Check convergence: IF $\left\Vert\lambda^{tr}-\lambda^{(k)} \right\Vert$ < $KTOL*\left\Vert\lambda^{(k)} \right\Vert$ EXIT |
| 1. $\lambda^{(k+1)}= \lambda^{tr}, k +=1$ |
| 1. Compute energy $G$_c_ and GOTO step 2 |

**Table D1:** Algorithm of contact mechanics solver.

As shown in the algorithm, our method uses two convergences criteria, ETOL and KTOL. The values are given 0.01 to ensure that the simulation reaches equilibrium and the penetration at the final step is sufficiently small, and thus guarantees the accuracy of the simulation. Meanwhile, the discretisation and linearisation are done according to [4].

**References**

1. Lang G, Lang GE. *Mechanics of swelling and damage in brain tissue: a theoretical approach* (Doctoral dissertation, Oxford University, UK).
2. Zhang X, Adamson RH, Curry FR, Weinbaum S. A 1-D model to explore the effects of tissue loading and tissue concentration gradients in the revised Starling principle. American Journal of Physiology-Heart and Circulatory Physiology. 2006 Dec;291(6):H2950-64.
3. Laursen TA, Maker BN. An augmented Lagrangian quasi‐Newton solver for constrained nonlinear finite element applications. International journal for numerical methods in engineering. 1995 Nov 15;38(21):3571-90.
4. Wriggers P. Computational contact mechanics. Laursen TA, editor. Berlin: Springer; 2006 Oct 6.
